# Effects of Modified Al_2_O_3_-Decorated Ionic Liquid on the Mechanical Properties and Impact Resistance of a Polyurethane Elastomer

**DOI:** 10.3390/ma14164712

**Published:** 2021-08-20

**Authors:** Fan Hu, Jun Gao, Biao Zhang, Fugang Qi, Nie Zhao, Xiaoping Ouyang

**Affiliations:** 1 School of Materials Science and Engineering, Xiangtan University, Xiangtan 411105, China; fancy123a@163.com (F.H.); 202021001699@smail.xtu.edu.cn (J.G.); zhaonie@xtu.edu.cn (N.Z.); oyxp2003@aliyun.com (X.O.); 2 Key Laboratory of Low Dimensional Materials and Application Technology of Ministry of Education, Xiangtan University, Xiangtan 411105, China

**Keywords:** impact resistance, ionic liquid, nano-Al_2_O_3_, polyurethane elastomer

## Abstract

In this work, a new composite material with excellent dynamic impact resistance and outstanding quasi-static mechanical properties was synthesized. The composite material is composed of a polyurethane elastomer and a novel nano-polymer. The nano-polymer was composed of silane coupling agent-modified alumina microspheres and functionalized ionic liquids by double bond polymerization. The universal testing machine and split Hopkinson pressure bar were used to characterize the compression behavior, strength and energy absorption of the composite materials under static and dynamic conditions. Additionally, the comprehensive mechanical properties of polyurethane elastomer with different nano-polymer loadings (0.5–2.5 wt.%) were studied. The results show that whether it was static compression or dynamic impact, the polyurethane elastomer with 1% nano-polymer had the best performance. For the composite material with the best properties, its compressive yield strength under the static compression was about 61.13% higher than that of the pure polyurethane elastomer, and its energy absorption of dynamic impacts was also increased by about 15.53%. Moreover, the shape memory effect was very good (shape recovery is approximately 95%), and the microscopic damage degree was relatively small. This shows that the composite material with the best properties can withstand high compression loads and high-speed impacts. The developed composite material is a promising one for materials science and engineering, especially for protection against compression and impacts.

## 1. Introduction

In recent decades, in military, industrial and civil settings, many unpredictable impact-damage attacks have occurred all over the world, such as low-speed extrusions and high-speed collisions. These events not only cause damage to vehicles, aircraft, ships and buildings, but also cause many casualties and property loss. It is urgent to improve the protective performance of engineering structures [1,2]. How to effectively resist the impact of an external attack on some target objects has always been a research hotspot for engineers and scholars in modern society. In recent years, researchers have paid more attention to the impact resistance and energy absorption properties of elastomer composite materials.

Polyurethane elastomer (PUE)-based composites are increasingly being considered promising materials in advanced protective structure fields, such as the space industry, superconducting engineering and so on, due to their advantages of high specific strength, good thermal characteristics, corrosion resistance, low shrinkage during curing and ease in processing [3,4,5,6]. Moreover, as a highly crosslinked thermosetting polymer material, PUE is widely used in various protective fields because of its ideal impact resistance, useful mechanical properties and excellent shape memory effect [7,8]. The main reason for the excellent impact properties of pure PUE can be explained as by its ability to undergo a transient phase transition under the condition of dynamic shock loading. When the pulse velocity is close to the respective segment’s mobility threshold, it will change from the rubber state to the leather state or even the glass state; thus, massive energy dissipation is achieved [9]. In addition, the unique resilience and toughness of PUE make the material have a high energy absorption capacity. However, with the continuous development of social and scientific technology, the compression resistance and impact resistance of PUE also need to be continuously improved. Therefore, the properties of PUE materials (such as the balance between high strength and high toughness, cyclic service performance, wear and corrosion resistance, etc.) should adapt to the new requirements of the continuous development of society through continued innovation and upgrading. 

As we all know, the one of the most important methods for improving the protective performance of a structure is developing and applying a new protective material. Moreover, the improvements to a material’s properties are mainly determined by the newly added materials with enhanced features. At present, the main method for improving the strength and impact resistance of materials is to prepare composite materials [10,11,12]. Nanoparticles are generally considered to be ideal materials, as they have good reinforcement properties due to their unique high strength and stiffness and “nano-effect” [13]. Among them, nano-Al_2_O_3_, which has good strength, high hardness and good wear resistance, is widely used to improve the materials’ mechanical properties. However, the enhancements of some specific properties (e.g., strength and impact resistance) are not only affected by the type of matrix system and the fillers, but also by the dispersion degree and the fillers’ adhesion of the matrix [14,15]. The addition of nano-Al_2_O_3_ is also usually accompanied by agglomeration [16] (specific surface area effect and strong van der Waals interactions). Therefore, the nanoparticles’ dispersion in the matrix of the composite material is a key factor that needs to be considered carefully [17].

The way to solve the problem of nanoparticle dispersion is usually to add surface modifications [18,19,20]. Meanwhile, ionic liquids (ILs) have become of interest during recent years, due to their physicochemical properties, such as structural designability, special cation and anion structure and high solubility [21,22,23]. They have been used in many fields, such as the synthesis and modification of polymeric materials and the processing of polymers [21,24]. By the way, the further addition of rigid nanofillers (such as nano-Al_2_O_3_) into polymer matrixes after incorporating soft modifiers (such as ILs) is an efficient way to further strengthen and toughen polymer-based composite materials. The concept has been well documented with many newly-emerging, high-performance composite materials containing multiscale and multifunctional fillers in the literatures [25,26,27,28,29]. Therefore, ILs can be designed to be grafted onto the surface of nano-Al_2_O_3_ to improve its dispersion in a matrix. In addition, the unique electrostatic effects of ILs can also enhance the dispersion ability of nanofillers [30]. These strengthening effects are mainly through the establishment of multi-interface synergistic effects, such as chemical bonding and electrostatic attraction. It is believed that the material can have multiple synergistic effects on strength and interface, thereby realizing mechanical and chemical interlocking, so that the material can effectively transfer stress and inhibit crack propagation when sustaining an impact. Logically, it can be foreseen that simultaneous modifications to the strength and toughness of nano-polymer-based composite materials in regard to high-speed impact conditions will be achieved by using modifiers of both rigid nanoparticles and soft ILs. Therefore, it is necessary to study the overall mechanical properties of PUE materials containing both nanofillers and ionic liquids under impact conditions. Elucidating the mechanism of reinforcement and toughening is critical for the design and manufacturing of a new generation of high-performance PUE-based composite materials for impact engineering. 

Finally, the above theory is based on actual engineering considerations. Taking all of these considerations into account, a new nano-polymer (IL@Al_2_O_3_) was synthesized and creatively applied to PUE (PUE-IL@Al_2_O_3_) to improve its strength and impact resistance in this paper. Based on the enhancement and toughening effects of nanoparticles and ILs, the energy absorption and destruction mechanism of PUE are elaborated. By exploring the influential factors such as material composition and ratio, we were able to create composites that can achieve compression resistance, impact resistance and good deformation recovery in engineering.

## 2. Materials and Methods

### 2.1. Materials

Al_2_O_3_ powders with an average particle size of 500 nm and 3-methacryloxypropyltrimethoxysilane (KH570, 99%) were purchased from Shanghai Aladdin Biochemical Technology Co., Ltd. (Shanghai, China) and Shanghai Macklin Biochemical Co., Ltd. (Shanghai, China) respectively, and were mainly used to prepare KH570 modified nano-Al_2_O_3_ (KH570-Al_2_O_3_). The reactants of IL and the initiator of the double bond polymerization reaction, 1-vinylimidazole (99%), 2-bromoethanol (96%) and 2,2-azobis (2-methylpropionitrile) (AIBN, 99%), were all purchased from Shanghai Macklin Biochemical Co., Ltd. (Shanghai, China). Other chemicals (analytical purity) used in this study were purchased from Hunan Huihong Reagent Co., Ltd (Changsha, China). The PUE materials were provided by Qingdao Green World New Material Technology Co., Ltd (Qingdao, China), which composed of two components: A (an isocyanate: –NCO, 6.212 wt.%) and B (a polyol: –OH, 6.358 wt.%).

### 2.2. Preparation of KH570 Modified Al_2_O_3_

First, 10.0 g of Al_2_O_3_ powder was dried at 80 °C for 24 h. After that, we added 2 g KH570 to the mixture of 60 mL of ethanol and 20 mL of H_2_O, whose pH had been adjusted to 4–5, and it had been mixed for about 30 min. Subsequently, 10.0 g of Al_2_O_3_ was added into the mixture, the reaction of the solution was ended after stirring at 60 °C for 6 h. The KH570 modified Al_2_O_3_ particles in the solution could be collected by centrifugation, washed several times with ethanol and then dried in a vacuum oven at 60 °C for 12 h.

### 2.3. Synthesis of IL

1-Vinylimidazole (9.41 g, 0.1 mol) and 2-bromoethanol (15.0 g, 0.12 mol) were added into a flask equipped with a magnetic stirrer. The mixture was refluxed to dark brown at 78 °C in a nitrogen atmosphere. By washing with diethyl ether to remove the unreacted starting materials, then drying in a vacuum oven at 60 °C for 12 h, a liquid of thick and dark brown oily products of [EOHVIm][Br] were obtained. As shown in Figure 4, ^1^H-NMR (400 MHz, DMSO, ppm), δ: 3.78 (t, 2H, –Im–CH_2_–), 4.28 (t, 2H, –CH_2_–O–), 5.46 (dd, 1H, =C–H), 6.02 (dd, 1H, =C–H), 7.36 (q, 1H, =CH–Im), 7.92 (s, 1H, C–CH–N of Im), 8.24 (s, 1H, N–CH–C of Im), 9.51 (s, 1H, N–CH–N of Im). The symbol Im refers to the imidazole ring.

### 2.4. Preparation of IL@Al_2_O_3_

**Figure 1:**
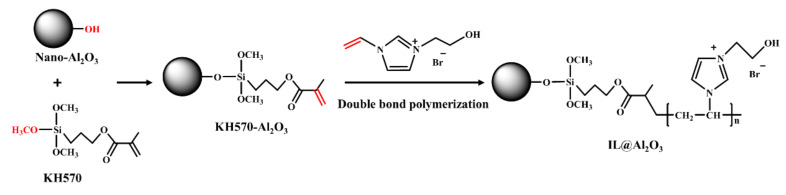
A schematic of the synthesis of IL@Al_2_O_3_.

First, 4.75 g IL was mixed with 0.25 g of dried KH570-Al_2_O_3_ in 20 mL of acetone with 0.005 g AIBN as the initiator. Under the protection of nitrogen, the reaction proceeded at 80 °C for 10 h. Then, the mixture dried in a vacuum oven at 60 °C for 12 h. The synthesis steps of IL@Al_2_O_3_ are shown in Figure 1.

### 2.5. Preparation of PUE-IL@Al_2_O_3_

The total molar ratio of isocyanate groups with respect to hydroxyl groups was maintained at 1:1.01. The formulations of the PUE-IL@Al_2_O_3_ are presented in Table 1. By mixing components A and B with a weight ratio of 5:2, IL@Al_2_O_3_ as stuffing was added to component B. After grinding, filler concentrations were 0.5, 1.0, 1.5, 2.0 and 2.5 wt.% of the PUE. Then came ultrasonic dispersion for 30 min, and the products were then mixed with component A evenly. The mixture was poured into a PTFE (polytetrafluoroethylene) mold with circle shapes at room temperature for 24 h, and the crosslinked PUE-IL@Al_2_O_3_ samples were finally obtained. To demonstrate the critical role of IL@Al_2_O_3_, a control sample only with Al_2_O_3_ (denoted as PUE-Al_2_O_3_) was prepared from similar steps. 

### 2.6. Structural Characterization

The FTIR spectra were collected by a Nicolet 6700 FTIR spectrometer (Thermo Fisher Scientific Inc., Massachusetts, USA) with a scanning range of 400–4000 cm^−1^. The ^1^H-NMR spectrum was used to characterize the successful synthesis and the purity of IL, which was collected by Bruker 400M (Bruker Corporation, Switzerland). The morphologies of Al_2_O_3_, KH570-Al_2_O_3_ were characterized by TEM (Titan G260–300) (FEI company, Hillsboro, OR, USA). The damaged surfaces of the PUE samples were observed under an optical microscope (Olympus BX53M) (Olympus Corporation, Tokyo, Japan).

### 2.7. Compression Mechanical Tests

**Figure 2:**
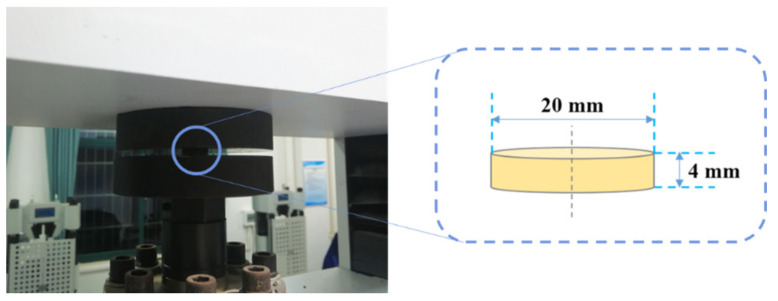
The quasi-static experimental device and the dimensions of each sample.

The quasi-static compression mechanical tests of PUE-IL@Al_2_O_3_ samples (Measure range: 0–10 kN) were carried out on a Shanghai Hualong universal testing machine (Shanghai, China), and each sample was compressed by 87.5% (3.5 mm) at a rate of 1.2 mm/min at room temperature. The reference standard for the experiment was ISO 604: 2002 [31]. The engineering stress–strain curves of PUE-IL@Al_2_O_3_ could be obtained after compression. The experimental device and dimensions of the samples for the mechanical tests are shown in Figure 2. 

### 2.8. Dynamic Impact Tests

**Figure 3:**
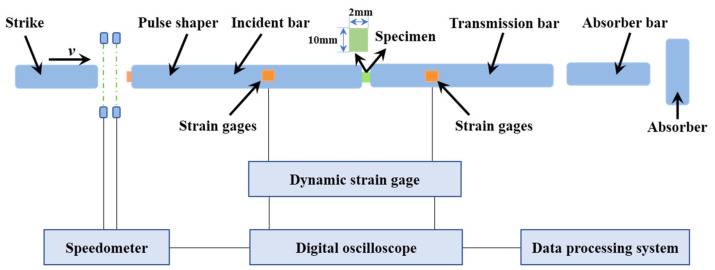
A sketch of the SHPB system.

In order to evaluate the impact resistance of the composite materials of PUE-IL@Al_2_O_3_, the impact experiment was carried out with an aluminum split Hopkinson pressure bar (SHPB) system (Changsha, China). The test platform device and schematic diagram are shown in Figure 3. The sample was clamped between the incident bar and the transmission bar, and high vacuum grease was also used to minimize friction at the interface between the bar ends and the sample. The SHPB device provided an almost constant impact velocity, which was used in this study to apply dynamic loadings to the PUE samples. The air gun launched the strike onto the incident bar, causing the impact bar to generate an elastic compression wave on the impact surface. The elastic compression wave moved along the incident rod toward the specimen. Due to the large differences in material impedance, the elastic compression wave had a phenomenon of differentiation. One part of the incident pulse (*ε_i_*) was reflected from the interface of the bar and sample—(*ε_r_*), and the other part was transmitted to the sample—the transmitted wave (*ε_t_*). 

According to 1D elastic wave propagation theory, the relationship [32] between the dynamic stress the dynamic strain and the strain rate can be shown as Equations (1)–(3):

(1) $$\sigma_{s}=\frac{E_{b}A_{b}}{A_{s}}\varepsilon_{t}$$

(2) $$\varepsilon_{s}=\frac{2C_{b}}{l_{s}}\int_{0}^{t}\left(\varepsilon_{i}-\varepsilon_{t}\right)dt$$

(3) $$\dot{\varepsilon_{s}}=\frac{2C_{b}}{l_{s}}\left(\varepsilon_{i}-\varepsilon_{t}\right)$$

The energy absorption capacity [33] of a sample was the integral of the stress and strain received during the impact failure process, which could be used to evaluate the impact resistance of the material or judge the brittleness and toughness of the material. It could be expressed by Equation (4):

(4) $$E_{A}=\int_{0}^{t}\sigma_{s}\cdot \varepsilon_{s}dt$$

where, $\sigma_{s}$, $\varepsilon_{s}$, $\dot{\varepsilon_{s}}$ and *E_A_* were the dynamic stress, the dynamic strain, the strain rate and the energy absorption capability, respectively; $\varepsilon_{i}$ and $\varepsilon_{t}$ were the incident pulse and transmitted pulse, respectively. $E_{b}$ was the elastic modulus of the bar material. $A_{s}$ and $A_{b}$ were the cross-sectional areas of the specimens and the bars, respectively. $C_{b}$ was the propagation velocity of the elastic wave in the bar; $l_{s}$ was the length of the sample.

## 3. Results

### 3.1. Structural Characterizations of KH570-Al_2_O_3_ and [EOHVIm][Br]

**Figure 4:**
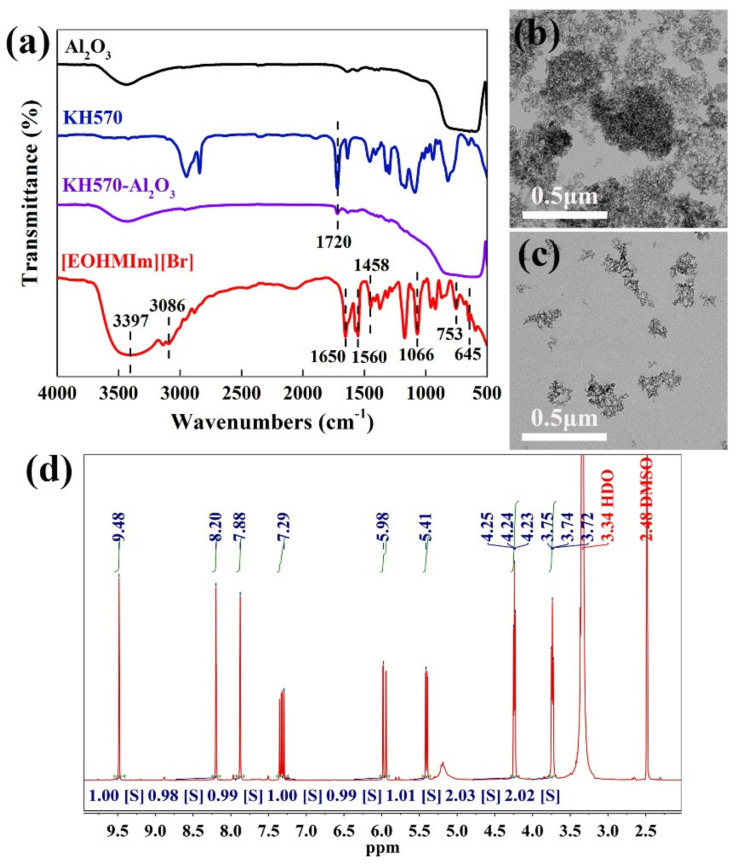
(**a**) The FTIR spectra of Al_2_O_3_, KH570, KH570-Al_2_O_3_ and [EOHVIm][Br]. TEM images of (**b**) Al_2_O_3_ and (**c**) KH570-Al_2_O_3_. (**d**) The ^1^H-NMR spectrum of the IL.

In order to explore the successful synthesis of KH570-Al_2_O_3_ and [EOHVIm][Br], FTIR, ^1^H-NMR and TEM tests were carried out. As shown in Figure 4a, by combining the infrared spectra of nano-Al_2_O_3_ and KH570, it was found that a new adsorption of C=O appeared in this spectrum at 1720 cm^−1^. In addition, the TEM images of Al_2_O_3_ and KH570-Al_2_O_3_ were investigated, as shown in Figure 4b,c. As can be seen, there were serious agglomerations and irregular morphology in the Al_2_O_3_. Furthermore, the image of KH570-Al_2_O_3_ shows single particles with a uniform size distribution, without agglomeration. Perhaps for this reason, the exposed –OH groups are replaced by organic functional groups, which reduces the number of active silicon hydroxyl groups and the tendency toward agglomeration. Based on the above data, it can be proved that KH570 was grafted onto the nano-Al_2_O_3_, and that KH570-Al_2_O_3_ powder has very good dispersibility.

The FTIR and ^1^H-NMR evidence about the successful synthesis of IL ([EOHVIm][Br]) is shown in Figure 4a,d. As can be seen from the Figure 4a, the peaks at around 3086, 1650, 1560, 1458 and 645 cm^−1^ correspond to the stretching vibrations of =C–H, C=C and C=N; the ring skeleton vibrations; and outer torus bending vibrations, respectively. Due to the intermolecular hydrogen bonding, a strong and wide absorption peak is present at about 3397 cm^−1^. The intermediate absorption peak at 1066 cm^−1^ corresponds to the stretching vibrations of a C–O bond. The intermediate absorption peak at 753 cm^−1^ corresponds to the out-of-plane bending vibrations of an O–H bond. Moreover, the additional peaks at about 3.74 and 4.24 ppm in the ^1^H-NMR spectrum also prove the successful synthesis of IL. In summary, characteristic absorption peaks appeared in the spectrum—enough to indicate the successful preparation of the [EOHVIm][Br]. 

### 3.2. Quasi-Static Properties of PUE-IL@Al_2_O_3_

**Figure 5:**
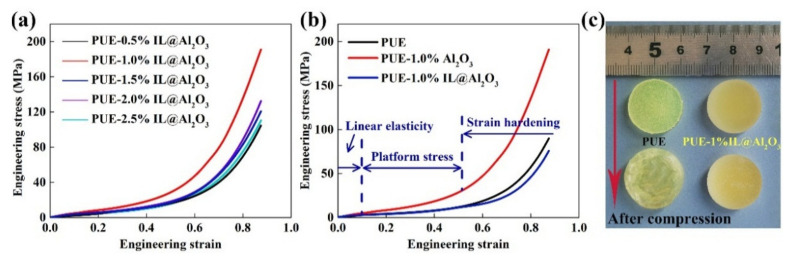
Quasi-static compression: (**a**) ER–ES curves of PUE-IL@Al_2_O_3_ with proportions from 0.5% to 2.5%; (**b**)ER–ES comparison curves; and (**c**) the compression deformation of PUE, PUE-1%Al_2_O_3_ and PUE-1%IL@Al_2_O_3_ specimens.

The static compression in the mechanical properties can reflect the compressive resistance of the material, so it is indispensable in the study of mechanical behavior. The compression details are shown and summarized in Figure 5 and Table 2. As can be seen in Figure 5a,b, the curves of the composite materials all have basically the same trend as pure PUE, and the curves generally go through three stages: initial linear elasticity, platform stress and a strain hardening region [34]. The peak stress in the initial linearly elastic region can be regarded as the compressive yield strength of a material, which is usually used to evaluate the compressive performance of a composite material [35]. 

As can be seen from Figure 5a, the 1% IL@Al_2_O_3_ had the best strengthening effect on the PUE. As the proportion increased, the strengthening effect decreased gradually, which can be interpreted as increasing nanofiller agglomeration in the matrix, as mentioned above. It can also be seen in Figure 5b that the compressive yield strength of PUE-1%Al_2_O_3_ (1.73 MPa) was worse than that of pure PUE (3.01 MPa). On this basis, the strengthening effect of having 1% IL@Al_2_O_3_ (compressive yield strength: 4.85 MPa, an increase of nearly 61.13%) is substantial. According to the phenomenon above, Figure 5c can also prove that PUE-1%IL@Al_2_O_3_ can not only withstand greater stress, but after the stress disappears, the material can almost completely return to the state before compression (the sample of PUE-1%Al_2_O_3_ was almost crushed, and could not be recycled). It can be explained that the PUE-1%IL@Al_2_O_3_ composite material is not only a kind of material with strong pressure resistance, but also one with an excellent shape memory effect.

### 3.3. Dynamic Impact Resistance of PUE-IL@Al_2_O_3_

**Figure 6:**
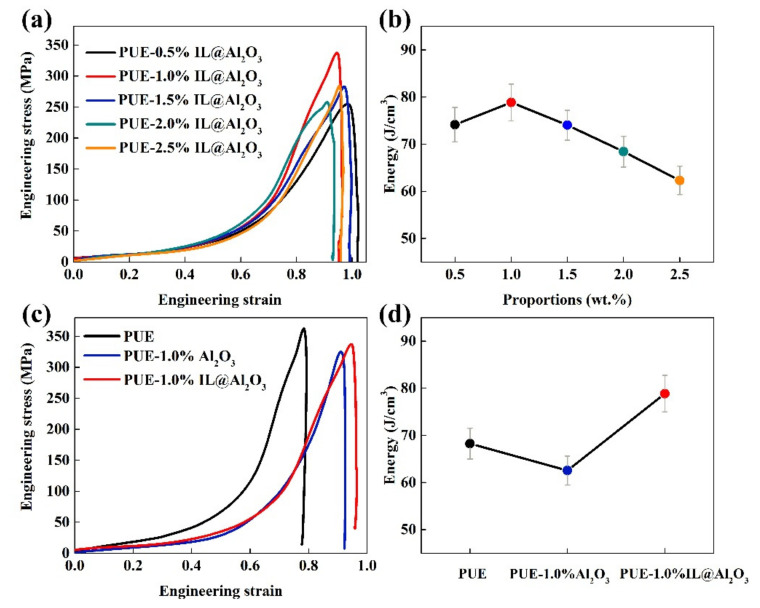
Dynamic impact: (**a**) ER–ES curves and (**b**) energy absorption curves of PUE with different proportions of IL@Al_2_O_3_ from 0.5% to 2.5% at the impact force of 0.2 MPa; (**c**) ER–ES curves and (**d**) energy absorption curves of different components (PUE, PUE-1%Al_2_O_3_, PUE-1%IL@Al_2_O_3_) at the impact force of 0.2 MPa.

Even though static compression can reflect the compressive performance of a material, the dynamic impact resistance of a material to high-speed impacts has become very important today. Therefore, this study also used SHPB to explore the dynamic mechanical properties of the material under high-speed impacts. By processing the three strain signals collected from the experiment of SHPB, the engineering stress–strain curves of the composite materials with different proportions and different components under the same strain rate (0.2 MPa) can be obtained, as shown in Figure 6. 

It can be seen from Figure 6 that the curves basically confirm the three stages mentioned above as static compression. However, there are many factors that affect the compressive yield strength under dynamic impacts, especially for relatively close values. Therefore, in the case of dynamic impacts, the energy absorption ability is a good basis for measuring the impact resistance of materials. Therefore, as shown in Figure 6b, the energy absorption of PUE with the addition of 1%IL@Al_2_O_3_ was still the best. As can be seen from Figure 6c,d, the curves show the engineering stress–strain and energy absorption changes of the PUE samples (different components) with filler content of 1% at the same impact force (0.2 MPa). Therefore, the energy absorption (approximately 78.85 J/cm^3^, improved by approximately 15.53%) of PUE-1%IL@Al_2_O_3_ is better than those of the other two groups. It can also be said that the impact resistance of PUE-1%IL@Al_2_O_3_ composite material is better than that the composite material of pure PUE and PUE-1%Al_2_O_3_.

**Figure 7:**
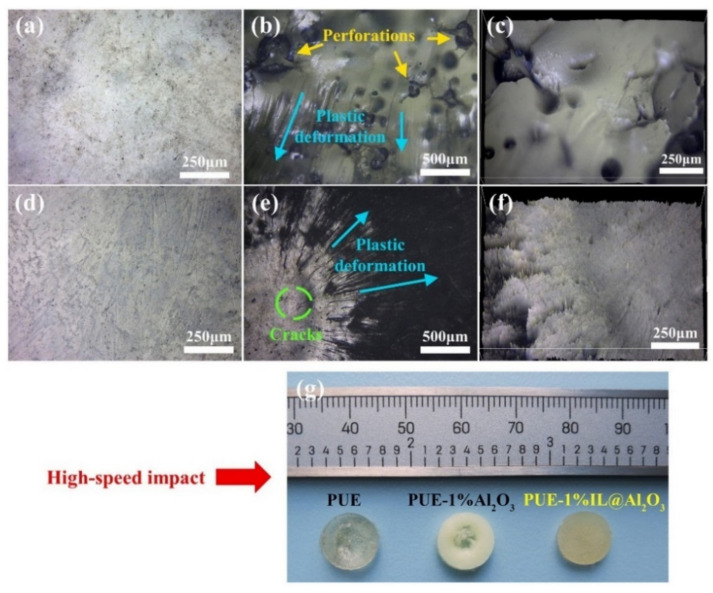
The pictures (**a**,**b**,**d**,**e**) show the damaged surfaces via optical microscopy; and (**c**,**f**) are the 3D images of the damaged surfaces. The pictures are of PUE (**a**–**c**) and PUE-1%IL@Al_2_O_3_ (**d**–**f**). The samples before the impact (**a**,**d**) and after impact (**b**,**c**,**e**,**f**). The picture of (**g**) is the sample’s appearance after impact.

Figure 7 compares the states of different composite materials before and after impact and the degrees of damage on the samples’ surfaces. As can be seen in Figure 7a,d, the sample surfaces before impact were basically smooth. After impact by the force of 0.2 MPa, the sample of PUE experienced irreversible bulge damage. At the microscopic level, as shown in Figure 7b,c, there were obvious cracks, perforations and plastic deformation zones on the sample’s surface. However, the sample of PUE-1%Al_2_O_3_ appeared to have almost perforated damage after the impact. It can be inferred from the above figures that the coalescence of micro-holes lead to the nucleation and propagation of cracks [36]. In Figure 7e,f, the picture and microscopic diagram of PUE-1%IL@Al_2_O_3_ are quite different from those of the previous two. There was almost no change in the appearance of PUE-1%IL@Al_2_O_3_. The surface damage to PUE-1%IL@Al_2_O_3_ was only small, and consisted of shallow cracks and many plastic deformation areas. 

## 4. Discussion

For the static compression test, the phenomenon described in Figure 5a,b can be explained as follows. First, it is well known that the PUE matrix and the reinforced nanoparticles (nano-Al_2_O_3_) are first elastically deformed to the yield point, and the nanoparticles first deform and yield with the load in the plastic deformation stage. Subsequently, when the deformation reaches the stress plateau area, many nanoparticles in the matrix will gradually break and fail. The failure of the nanoparticles will be accompanied by the transfer of the load to the matrix, resulting in a sharp increase in stress [37,38]. Apparently, the fillers can strengthen the matrix mainly because of its dispersibility and performance. Therefore, the characteristics and loadings of the filler are important factors that can affect the mechanical properties of the material [17]. From the phenomena produced by PUE-1%Al_2_O_3_, it can be concluded that the rigid Al_2_O_3_ nanoparticles have a certain strengthening effect on the PUE matrix sometimes. However, inappropriate proportion (PUE-1%Al_2_O_3_) and agglomeration phenomena caused by high specific surface energy usually still lead to a decrease in the performance of the composite material. The positive effects of adding nanoparticles on the hardness and compressive strength of the matrix have been confirmed before. Similarly, inappropriate proportions of nanoparticles and high cohesive energy will increase the brittleness of the material and the agglomeration of nanoparticles [17]. Particularly, the compression resistance of PUE-1%IL@Al_2_O_3_ is much better than those of the other two groups, which may be because the IL can improve the dispersion of nano-Al_2_O_3_ particles to a certain extent, and nano-Al_2_O_3_ has a certain connection with PUE matrix molecules (A component: –NCO) through the grafted functional groups of IL (–OH). Therefore, the nano-Al_2_O_3_ can drive the surrounding matrix into the strain hardening domain when the sample is impacted [39].

For the dynamic impact test, we can infer the following reasons from the description in Section 3.3. On the one hand, the improvements in the properties of the PUE-1%IL@Al_2_O_3_ composite are mainly due to the formation of an organic–inorganic interface with the PUE matrix. There was good compatibility between the inorganic nanoparticles and the PUE matrix through the connection with IL. On the other hand, since IL itself has hydrogen bonds and cations and ions, when it is impacted by the external force, the energy consumption caused by the breakage of hydrogen bonds and the relative slip between cations and ions can offset some of the external forces, resulting in increased energy absorption of PUE materials. It is worth mentioning that the strain hardening region of the PUE-IL@Al_2_O_3_ is delayed. That can be explained by the fact that the addition of IL can make the composite material fully plasticized [36]. Therefore, the strain elongation of the PUE-IL@Al_2_O_3_ composite material has increased. From the above analysis of the results, it can be concluded that the addition of IL will reduce the strength of composite materials, thereby affecting the mechanical properties of composite materials. Our results show that the combination of nanoparticles and IL led to synergistic effects. The combination significantly improves toughness and energy absorption [40,41,42]. The PUE-IL@Al_2_O_3_ has greater deformability to cater the impact than PUE under the same engineering stress. The phenomenon of the deformation may be due to the “thermal effect” generated by the composite materials at high impact force. As shown in Figure 7, the picture of the samples’ surfaces before and after the impact can also prove the above statement. In short, the main damage and energy consumption modes are mainly plastic deformation with a certain degree of recovery.

**Figure 8:**
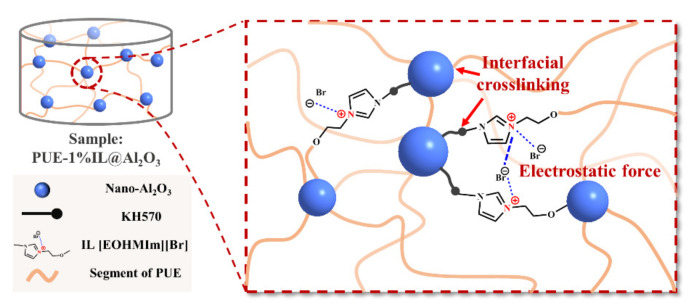
An inner structure diagram of the specimen of composite material (PUE-1%IL@Al_2_O_3_).

In conclusion, the above phenomena indicate that the performance improvement of PUE-IL@Al_2_O_3_ under high-speed impact is mainly due to the following points, as shown in Figure 8: (1) the strength of the rigid nano-Al_2_O_3_ can offset part of the impact force, but the nanoparticles are easy to agglomerate in the matrix, which causes stress concentration and becomes the defect in the matrix [43]; (2) the IL can not only modify nano-Al_2_O_3_ through double bond polymerization to improve the dispersion of nanoparticles, but also improve its interfacial cross-linking and adhesion effect [35] in regard to nano-Al_2_O_3_ and the PUE matrix through the reaction of [EOHVIm][Br] (–OH) and the chemical group of the A component (–NCO) of the PUE. In addition, the IL, which provides the special effects such as electrostatic force [44] and hydrogen bonding [45], can strengthen the connection of the PUE and IL@Al_2_O_3_, so that the composite material exhibits increases in hardness and strength. Additionally, the IL@Al_2_O_3_ has good dispersion in the PUE matrix, which can be explained as a strong interfacial cross-linking force between organic polymers and inorganic nanoparticles. (3) PUE-IL@Al_2_O_3_ has good deformation recovery. By generating heat and plastic deformation during impact, part of the energy can be consumed, and the generation of cracks in the matrix can be reduced [17]. In conclusion, the synchronous strengthening of the strength and toughness of PUE-IL@Al_2_O_3_ composite material are mainly attributed to the chemical bonding multiscale interface established by self-assembled hybrid particles, which combines the micro-scale toughening effect of IL with the bridging effect of rigid nanoparticles and nanoparticles. In summary, only 1% filler content of IL@Al_2_O_3_ added to the PUE provided excellent strength and energy absorption. Therefore, as a composite material with high strength, high hardness, high impact resistance, high energy absorption and low cost, it is expected to be widely used in protective materials and structures.

## 5. Conclusions

This research creatively adopted the chemical modification method of double bond polymerization to graft functional IL onto the surfaces of nanoparticles to prepare a novel nano-polymer. The novel nano-polymer was prepared via double bond polymerization between KH570-Al_2_O_3_ and a designed IL ([EOHMIm][Br]) with special functional groups (C=C and ‒OH). The nano-polymer was creatively applied to a PUE matrix to prepare a new type of high-strength and impact-resistant composite material (PUE-IL@Al_2_O_3_). The research mainly used a combination of dynamic and static compression testing to analyze the mechanical behavior of and damage to composite materials under high-strength compression and high-speed impacts. The main conclusions are as follows:

(1) From the static compression curves, the PUE-IL@Al_2_O_3_ with 1% IL@Al_2_O_3_ could withstand the maximum ultimate peak stress, which is an increase of about 61.13% compared with the pure PUE. Moreover, the compressed specimen had good morphological after unloading.
(2) According to the dynamic impact curve after the high impact force (0.2 MPa), the impact resistance (evaluated by energy absorption) was increased by about 15.36%. Moreover, the deformation recovery of the sample is excellent (approximately 95%). The improvements of the strength and energy absorption of composite material mainly depend on the synergistic enhancement of nano-Al_2_O_3_ and IL and the synergistic effects of multiple interface permeations.

The results show that the new composite material (PUE-IL@Al_2_O_3_) not only has high compression strength and excellent impact resistance, but also has outstanding shape memory. The PUE-based composite material prepared in this study has compression resistance, impact resistance, durability and good recovery, so could be used in many engineering applications. To be sure, the results obtained in this study will bring great significance to practical applications. First, the preparation method was simple and economical. Secondly, this study not only used a new nano-filler modification method, but also prepared composite materials by only adding fillers, which greatly improved the properties of the PUE matrix material. It can be seen that for the rapidly developing civil and military engineering fields, continuously improving compression and impact resistance will be indispensable. New requirements and ideas will be put forward for impact resistant materials. It is desirable to prepare an upgraded material which not only has high impact resistance, but also has high repairability, so that it can withstand higher and repeated impacts.

## Figures and Tables

**Table 1:** Formulations of PUE-IL@Al_2_O_3_.

Sample	Relative Mole Ratio A(–NCO):B(–OH):IL@Al_2_O_3_	IL@Al_2_O_3_ Content (wt.%)
PUE-0.5%IL@Al_2_O_3_	1:0.99:0.02	0.5
PUE-1.0%IL@Al_2_O_3_	1:0.97:0.04	1.0
PUE-1.5%IL@Al_2_O_3_	1:0.95:0.06	1.5
PUE-2.0%IL@Al_2_O_3_	1:0.93:0.08	2.0
PUE-2.5%IL@Al_2_O_3_	1:0.91:0.10	2.5

**Table 2:** A data summary for PUE, PUE-Al_2_O_3_ and PUE-IL@Al_2_O_3_ under quasi-static compression testing.

Sample	Maximum Force (kN)	Elastic Modulus (MPa)	Maximum Strength (MPa)	Compression Rate (%)
PUE	28.34	95.8	90.2	48.76
PUE-1.0%Al_2_O_3_	21.24	57.0	67.6	58.16
PUE-0.5%IL@Al_2_O_3_	32.90	97.4	104.7	54.52
PUE-1.0%IL@Al_2_O_3_	60.32	210.2	192.0	51.14
PUE-1.5%IL@Al_2_O_3_	44.11	161.8	140.37	48.93
PUE-2.0%IL@Al_2_O_3_	41.61	140.2	132.5	49.99
PUE-2.5%IL@Al_2_O_3_	34.72	119.8	110.5	50.22

## Data Availability

Not applicable.
